# Long-Range Source Localization in the Deep Sea Using Adaptive FDSL with a Few-Element Array

**DOI:** 10.3390/s26051495

**Published:** 2026-02-27

**Authors:** Jingwen Yin, Haklim Ko, Hojun Lee

**Affiliations:** Department of Information and Communication Engineering, Hoseo University, Asan 31499, Republic of Korea

**Keywords:** deep sea, few elements, frequency difference source localization (FDSL), long-range source localization, matched field processing (MFP), minimum variance distortionless response (MVDR), multiple signal classification (MUSIC)

## Abstract

Matched Field Processing (MFP) suffers from environmental mismatch in deep-sea long-range source localization. Although Frequency Difference Matched Field Processing (FDMFP) improves mismatch tolerance, it fails due to caustic phase effects. Frequency Difference Source Localization (FDSL) effectively compensates for caustic phase errors by applying frequency-difference processing to both the measured field and the replica field. However, conventional FDSL typically relies on large-aperture arrays with numerous elements, resulting in high deployment costs and bulky systems. Furthermore, it exhibits limited resolution and elevated sidelobes. These limitations are exacerbated under reduced element counts and low signal-to-noise ratio (SNR) conditions. To improve performance under low SNR and small-array configurations, this paper proposes the FDSL-MVDR and FDSL-MUSIC methods by deriving adaptive weight vectors based on the frequency-difference covariance structure and redefining the ambiguity surface. Numerical simulations in a deep-sea Munk environment (source range 195 km, depth 1000 m) using a 15-element vertical line array demonstrate that the adaptive FDSL methods outperform conventional FDSL in terms of peak sharpness and sidelobe suppression. FDSL-MUSIC achieves approximately 100% localization success at SNR = −5 dB, a 4 dB improvement over conventional FDSL. Performance analyses under representative environmental mismatches indicate that the adaptive FDSL methods maintain robust localization performance and high-resolution characteristics in complex deep-sea environments. These results validate the feasibility of high-precision deep-sea localization using a few-element array.

## 1. Introduction

Deep-sea long-range passive source localization plays a critical role in ocean surveillance, underwater target tracking, and marine environmental monitoring. As a localization method that leverages a priori knowledge of ocean waveguide environments, Matched Field Processing (MFP) estimates the source position by quantifying the correlation between measured acoustic fields and theoretical replica fields [1,2,3,4,5]. However, MFP is highly susceptible to environmental mismatch, with its performance severely degraded by ocean ambient noise, receiver array sparsity, and uncertainties in environmental parameters, ultimately leading to substantial deterioration in localization accuracy or even complete failure.

To mitigate the impact of environmental mismatch on localization performance, Worthmann et al. introduced Frequency Difference Matched Field Processing (FDMFP) [6,7,8,9,10]. This approach transforms the problem from high-frequency to low-frequency processing by applying frequency-difference processing to received data, subsequently performing matched field processing in the low-frequency domain. Experimental validation demonstrates that FDMFP exhibits enhanced tolerance to environmental mismatch in shallow water waveguide environments, yielding more robust source localization performance compared to conventional MFP.

However, the application of FDMFP in deep-sea long-range scenarios is fundamentally constrained. In deep-sea environments, acoustic waves undergo complex multipath propagation, with sound rays producing caustics in the water column, thereby inducing significant caustic phase distortions. These distortions prevent FDMFP from accurately establishing the correlation between measured and replica fields, consequently causing localization failure. To address this critical limitation, Dowling et al. substantially advanced FDMFP by proposing Frequency Difference Source Localization (FDSL), which was successfully validated using experimental data from the Philippine Sea. The key innovation of FDSL lies not only in applying frequency-difference processing to the received signals but, more importantly, in applying the same processing to the replica fields, thereby effectively compensating for caustic phase errors and attaining robust localization performance in deep-sea long-range propagation scenarios [11,12,13].

Despite demonstrating promising application potential, existing FDSL research has primarily been conducted under idealized experimental configurations. For instance, Dowling et al.’s Philippine Sea experiment employed a full-depth vertical line array comprising 149 hydrophone elements, with data acquired under high signal-to-noise ratio (SNR) conditions of approximately 20 dB [11]. However, in practical ocean applications, receiving array systems are often constrained by deployment costs, physical space limitations, and technical implementation complexity, making the deployment of large-aperture, high-density array configurations impractical. Simultaneously, realistic ocean environments ubiquitously exhibit complex ambient noise and dynamic interference, resulting in received SNRs substantially lower than ideal conditions. Consequently, investigating high-performance source localization under non-ideal conditions characterized by small apertures, limited array elements, and low SNRs is of significant importance for practical engineering applications.

Moreover, despite its computational efficiency, conventional FDSL suffers from constrained resolution and elevated sidelobe levels under low SNR and few-element array configurations. These limitations significantly diminish the prominence of the main peak against the background level.

In contrast, adaptive beamforming techniques, such as Minimum Variance Distortionless Response (MVDR) and Multiple Signal Classification (MUSIC), offer the potential for superior sidelobe suppression and resolution enhancement. Accordingly, this paper establishes the FDSL-MVDR and FDSL-MUSIC methods by formulating adaptive weight vectors based on the frequency-difference covariance matrix and redefining the ambiguity surface.

The main contributions of this study are summarized as follows:

(1) Methodological Formulation: This study proposes the FDSL-MVDR and FDSL-MUSIC methods by deriving the adaptive weighting vectors and redefining the ambiguity surface within the frequency-difference domain. These methods enable super-resolution and superior sidelobe suppression, effectively overcoming the resolution limitations of conventional FDSL.
(2) Performance Assessment in Matched Environments: Through systematic Monte Carlo simulations conducted in a deep-sea Munk environment, the proposed methods are evaluated using a 15-element vertical array across a wide SNR range (−10 dB to 5 dB). The analysis demonstrates that FDSL-MUSIC achieves a localization success rate of approximately 100% at an SNR of −5 dB, validating the feasibility of high-precision localization using cost-effective, small-aperture arrays.
(3) Robustness Assessment in Mismatched Environments: Moving beyond the matched case, this paper rigorously evaluates the robustness of the proposed methods under mismatched scenarios. Specifically, the impact of four typical environmental uncertainties is investigated: mismatches in sound speed profile (SSP), water depth, seabed attenuation, and array element position. The results demonstrate that the proposed adaptive methods maintain stable localization performance under mismatched deep-sea conditions, effectively preserving the inherent environmental robustness of the conventional FDSL method.

The remainder of this paper is organized as follows. Section 2 introduces the theoretical background of conventional MFP and FDMFP. Section 3 details the derivation of the proposed adaptive FDSL methods. Section 4 presents the simulation results, providing a comprehensive evaluation of localization accuracy, resolution capability, and robustness against environmental uncertainties. Section 5 concludes the paper.

## 2. Theoretical Background

In this paper, all employed source localization methods can be regarded as extended forms of MFP.

### 2.1. MFP

**Figure 1:**
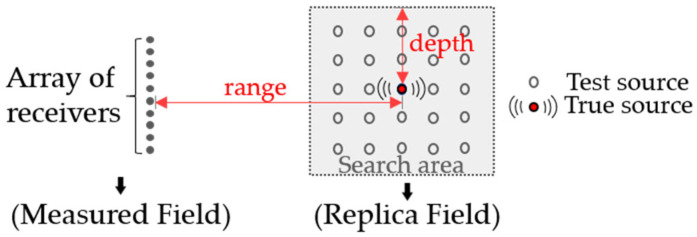
Schematic diagram of MFP.

MFP can be viewed as a generalization of beamforming, extending its applicability from free-space propagation to underwater waveguide environments by incorporating knowledge of the acoustic channel. Figure 1 shows a schematic of MFP, where a true source and a hydrophone array are deployed in the ocean waveguide.

The MFP localization procedure can be broadly divided into three steps. First, acoustic signals are received by the array and are defined as the measured field. Second, the search region is discretized into $N_{z}\times N_{r}$ grid points, where $N_{z}$ and $N_{r}$ denote the numbers of grid points in depth and range, respectively. Assuming a test source exists at a particular grid point, the theoretical acoustic pressure field at the array is calculated by combining the waveguide environmental parameters with the acoustic propagation model. This calculated field is termed the replica field. Finally, the correlation between the measured field and the replica field is computed for that grid point. By scanning through all grid points, an $N_{z}\times N_{r}$ matrix is obtained, where the position corresponding to the maximum correlation value represents the estimated source location. In summary, the conventional MFP method determines the source position by computing the correlation between the measured field and the replica field.

### 2.2. FDMFP

Measured Field: Consider a narrowband signal at frequency ω received by an M-element array. The received data in the frequency domain can be written as

(1) P(ω)=s(ω)g(ω)+n(ω)

where $P(\omega )={\left[p_{1}(\omega ),p_{2}(\omega ),\ldots ,p_{M}(\omega )\right]}^{T}$ represents the frequency-domain complex acoustic pressure vector, $s\left(\omega \right)$ represents the frequency-domain source signal, $g(\omega )={\left[g_{1}(\omega ),g_{2}(\omega ),\ldots ,g_{M}(\omega )\right]}^{T}$ is the Green’s function vector derived from the Helmholtz equation, and n(ω) is complex Gaussian white noise.

By applying frequency-difference processing to the received data, the high-frequency content is shifted to a lower-frequency difference component, forming the frequency-difference signal. The frequency-difference signal is the conjugate product of pressure fields at two different frequency components, also known as the frequency-difference autoproduct. From the perspective of signal construction, frequency-difference processing requires that the source signal has a certain bandwidth. For illustration, consider a dual-frequency signal, where the source signal consists of two frequency components, ω+∆ω/2 and ω−∆ω/2, respectively. The frequency-difference signal is expressed by the following equation [14,15,16,17].

(2) $$\mathrm{AP}(\omega ,∆\omega )=P(\omega +\frac{∆\omega }{2})P^{*}(\omega -\frac{∆\omega }{2})$$

where ∆ω is the difference frequency between the two components, and the ${\left(\cdot \right)}^{*}$ denotes complex conjugation.

By substituting the ray-path multipath expansion of the acoustic pressure field, $p_{j}\left(\omega \right)=\sum_{x=1}^{X}A_{jx}exp(i\omega \tau_{jx})$, into the autoproduct definition in (2), the frequency-difference signal ${AP}_{j}(\omega ,∆\omega )$ at the j-th hydrophone is decomposed into diagonal terms and cross terms. Here, X denotes the total number of multipaths, while $A_{jx}$ and $\tau_{jx}$ represent the amplitude and time delay of the x-th propagation path to the j-th hydrophone, respectively. The expansion is expressed as

(3) $$\begin{array}{cc} {AP}_{j}\left(\omega ,∆\omega \right) & =\sum_{x=1}^{X}{\left|A_{jx}\right|}^{2}\exp\left(i∆\omega \tau_{jx}\right)\\ & +\sum_{x\neq y}^{X,X}A_{jx}A_{jy}^{*}exp(i\frac{∆\omega }{2}(\tau_{jx}+\tau_{jy}))exp(i\omega (\tau_{jx}-\tau_{jy})) \end{array}$$

The diagonal terms (*x* = *y*) constitute the desired signal component, as their phase depends solely on the difference frequency ∆ω, which is equivalent to a conventional low-frequency acoustic signal. In contrast, the cross terms (x≠y) are undesirable components. Since their phase is modulated by the high-frequency carrier ω, the cross terms fluctuate rapidly as ω varies. In multipath environments, the presence of these oscillatory cross terms reduces the similarity between the frequency-difference signal and the low-frequency replica field, thereby degrading localization accuracy.

The cross-spectral density matrix (CSDM) for a single frequency-difference signal is defined as

(4) $$R(\omega ,∆\omega )=\frac{\mathrm{AP}(\omega ,∆\omega ){\mathrm{AP}}^{H}(\omega ,∆\omega )}{{\mathrm{AP}}^{H}(\omega ,∆\omega )\mathrm{AP}(\omega ,∆\omega )}$$

To mitigate the influence of cross terms, bandwidth averaging is applied by computing the CSDM from multiple frequency-difference signals:

(5) $$\bar{R}\left(\omega ,∆\omega \right)=\frac{1}{K}\sum_{k=1}^{K}\frac{{\mathrm{AP}}_{k}(\omega ,∆\omega ){\mathrm{AP}}_{k}^{H}(\omega ,∆\omega )}{{\mathrm{AP}}_{k}^{H}(\omega ,∆\omega ){\mathrm{AP}}_{k}(\omega ,∆\omega )}$$

where K denotes the number of frequency-difference signal realizations constructed from the source signal bandwidth with the same frequency ∆ω. This value is determined by the signal bandwidth $\omega_{BW}$ and the frequency resolution δω, calculated as $K=(\omega_{BW}-∆\omega )/\delta \omega +1$.

Replica Field: For a test location $x_{t}=(r_{t},z_{t})$, where $r_{t}$ is the source–array horizontal range and $z_{t}$ is the source depth, the Green’s function $g(x_{t},\omega )$ from the source to the receiving array is obtained using the KRAKEN normal-mode model [18,19]. The normalized MFP replica field vector is then given by (6).

(6) $$w_{\mathrm{MFP}}\left(x_{t},\omega \right)=\frac{g\left(x_{t},\omega \right)}{\left\|g\left(x_{t},\omega \right)\right\|}$$

The FDMFP replica field vector is similar to that of MFP, except that the original signal frequency ω is replaced by the difference frequency value ∆ω, as shown in (7).

(7) $$w_{FDMFP}\left(x_{t},∆\omega \right)=\frac{g\left(x_{t},∆\omega \right)}{\left\|g\left(x_{t},∆\omega \right)\right\|}$$

Matching processing: By correlating the measured field with the replica field, the FDMFP localization ambiguity surface is obtained as

(8) $$B_{\mathrm{FDMFP}}\left(x_{t}\right)=w_{\mathrm{FDMFP}}^{H}\left(x_{t},∆\omega \right)\bar{R}\left(\omega ,∆\omega \right)w_{\mathrm{FDMFP}}\left(x_{t},∆\omega \right)$$

## 3. Adaptive FDSL

**Figure 2:**
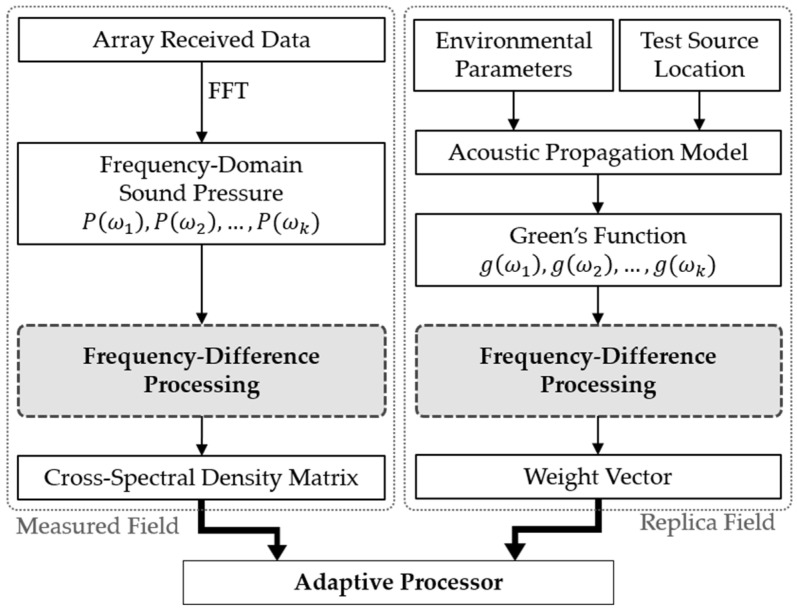
Schematic diagram of the proposed adaptive FDSL methods.

FDSL, as an advancement of FDMFP, introduces a unique replica field structure by applying frequency-difference processing to both the measured and replica fields, thereby effectively correcting caustic phase errors. However, conventional FDSL suffers from constrained resolution and elevated sidelobe levels, particularly under low SNR and few-element array configurations. To address these limitations, this paper proposes the FDSL-MVDR and FDSL-MUSIC methods. By formulating adaptive weight vectors based on the frequency-difference covariance matrix, the proposed methods achieve superior resolution and sidelobe suppression compared to conventional FDSL. The schematic diagram of the proposed methods is illustrated in Figure 2. Both the array received data and the replica field undergo frequency-difference processing to extract the difference-frequency components. These are then used to formulate the data covariance matrix and the replica weight vector, respectively.

Although the frequency-difference signal exhibits reduced multipath complexity similar to a low-frequency signal, a mismatch persists between the generated difference-frequency field and the ideal low-frequency field. Therefore, the FDSL replica field must also undergo frequency-difference processing. The FDSL replica field vector is expressed by (9).

(9) $$w_{\mathrm{FDSL}}\left(x_{t},∆\omega \right)=g\left(x_{t},\omega +\frac{∆\omega }{2}\right)g^{*}\left(x_{t},\omega -\frac{∆\omega }{2}\right)$$

Similarly, consistent with the measured field processing in (5), bandwidth averaging over the same number of realizations K is applied to the replica field, as shown in (10).

(10) $${\bar{w}}_{\mathrm{FDSL}}\left(x_{t},∆\omega \right)=\frac{1}{K}\sum_{k=1}^{K}g\left(x_{t},\omega +\frac{∆\omega }{2}\right)g^{*}\left(x_{t},\omega -\frac{∆\omega }{2}\right)$$

Subsequently, the bandwidth-averaged replica field ${\bar{w}}_{FDSL}$ is normalized to obtain the final FDSL replica field vector, which can be expressed as (11).

(11) $$w(x_{t},∆\omega )=\frac{{\bar{w}}_{\mathrm{FDSL}}(x_{t},∆\omega )}{\left\|{\bar{w}}_{\mathrm{FDSL}}(x_{t},∆\omega )\right\|}$$

**Figure 3:**
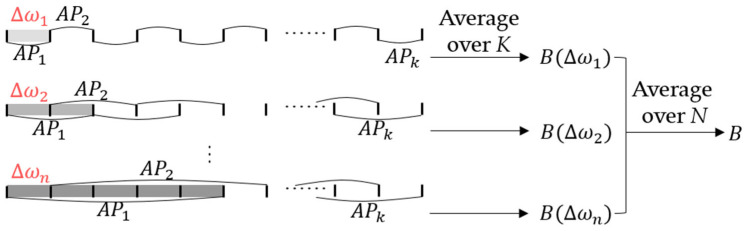
Schematic of two-stage averaging strategy for FDSL with multiple difference frequency values.

By correlating the measured field with the replica field, the FDSL localization ambiguity surface is obtained as shown in (12).

(12) $$B_{FDSL}\left(x_{t}\right)=\frac{1}{N}\sum_{n=1}^{N}w^{H}\left(x_{t},∆\omega_{n}\right)\bar{R}\left(\omega ,∆\omega_{n}\right)w\left(x_{t},∆\omega_{n}\right)$$

where N denotes the number of selected difference frequency values. The correlation values calculated for all grid points constitute the ambiguity surface, where the position of the maximum value corresponds to the estimated source location. A two-stage averaging strategy is implemented to enhance the localization robustness. First, for each fixed-difference frequency value $∆\omega_{n}$, bandwidth averaging is performed over K frequency-difference signals as shown in (5) and (10), which reduces the influence of cross terms. Second, the ambiguity surfaces obtained from N different difference frequency values $∆\omega_{n}$ are averaged incoherently as indicated by (12), which further enhances robustness. The two-stage averaging strategy for FDSL with multiple difference frequency values is illustrated in Figure 3. The difference frequency values $∆\omega_{n}$ are constrained within the signal bandwidth $\omega_{BW}$, satisfying $0<∆\omega_{n}\leq \omega_{BW}.$

### 3.1. FDSL-MVDR

The FDSL-MVDR method is formulated by applying the Minimum Variance Distortionless Response (MVDR) criterion to the frequency-difference signal. The fundamental principle is to minimize the total output power of the frequency-difference field to suppress noise and interference while maintaining a distortionless response for the signal originating from the test source location [20,21,22,23]. This constrained optimization problem is expressed as (13).

(13) $$\underset{w_{\mathrm{MVDR}}}{\min}w_{\mathrm{MVDR}}^{H}\bar{R}w_{\mathrm{MVDR}}\;subject\;to\;w_{\mathrm{MVDR}}^{H}w\left(x_{t},∆\omega \right)=1$$

where $w_{\mathrm{MVDR}}$ denotes the adaptive weight vector, $w\left(x_{t},∆\omega \right)$ is the M×1 dimensional replica field vector, and $\bar{R}$ is the CSDM of the frequency-difference signal.

The optimal weight vector is derived by solving this optimization problem using the Lagrange multiplier method, as shown in (14).

(14) $$w_{\mathrm{MVDR}}=\frac{{\bar{R}}^{-1}w\left(x_{t},∆\omega \right)}{w^{H}\left(x_{t},∆\omega \right){\bar{R}}^{-1}w\left(x_{t},∆\omega \right)}$$

The localization ambiguity surface for FDSL-MVDR is obtained by incoherently averaging over N difference frequency values, as given by (15).

(15) $$B_{\mathrm{FDSL}\text{-}\mathrm{MVDR}}\left(x_{t}\right)=\frac{1}{N}\sum_{n=1}^{N}\frac{1}{w^{H}\left(x_{t},∆\omega_{n}\right){\bar{R}}^{-1}w\left(x_{t},∆\omega_{n}\right)}$$

### 3.2. FDSL-MUSIC

The FDSL-MUSIC method is developed by exploiting the orthogonality between the signal and noise subspaces of the frequency-difference field to achieve super-resolution estimation. Unlike conventional beamforming, this method overcomes the Rayleigh resolution limit and produces sharper spectral peaks [24,25,26].

Assuming D uncorrelated sources and spatially white Gaussian noise with variance $\sigma^{2}$, the M × M CSDM of the frequency-difference field can be written as

(16) $$\bar{R}(\omega ,\Delta \omega )=W(\omega ,\Delta \omega )R_{s}W^{H}(\omega ,\Delta \omega )+\sigma^{2}I$$

where $W(\omega ,\Delta \omega )=[w(x_{t,1},∆\omega ),\ldots ,w(x_{t,D},∆\omega )]\in C^{M\times D}$ is the FDSL replica field matrix for D source locations, $R_{s}$ is the signal covariance matrix, and $I\in C^{M\times M}$ is the identity matrix.

The eigenvalue decomposition of $\bar{R}(\omega ,\Delta \omega )$ is given by

(17) $$\bar{R}(\omega ,\Delta \omega )=U\Lambda U^{H}=\sum_{i=1}^{M}\lambda_{i}e_{i}e_{i}^{H}$$

where $\Lambda =diag(\lambda_{1},\lambda_{2},\ldots ,\lambda_{M})$ denotes the diagonal matrix of eigenvalues, and $U=[e_{1},e_{2},\ldots ,e_{M}]$ is the eigenvector matrix.

Sorting the eigenvalues in descending order, the eigenvectors $\left\{e_{1},\ldots ,e_{D}\right\}$ corresponding to the D largest eigenvalues span the signal subspace $E_{s}.$ The remaining M−D eigenvectors span the noise subspace $E_{n}$. By definition, the noise subspace is orthogonal to the signal subspace, $E_{s}⊥E_{n}$. The signal and noise subspaces are defined as

(18) $$E_{s}=[e_{1},e_{2},\ldots ,e_{D}]\in C^{M\times D}$$

(19) $$E_{n}=[e_{D+1},e_{D+2},\ldots ,e_{M}]\in C^{M\times (M-D)}$$

The number of sources D can be estimated using model order selection methods such as the Akaike Information Criterion (AIC) and the Minimum Description Length (MDL) [27]. Any FDSL replica field vector $w\left(x_{t},∆\omega \right)$ corresponding to a true source location must be orthogonal to the noise subspace. To measure this orthogonality, a squared norm is defined:

(20) $$d^{2}={\left\|E_{n}^{H}w\left(x_{t},∆\omega \right)\right\|}^{2}=w^{H}\left(x_{t},∆\omega \right)E_{n}E_{n}^{H}w\left(x_{t},∆\omega \right)$$

Taking the reciprocal of the squared norm expression creates sharp peaks at the true source locations. When $w\left(x_{t},∆\omega \right)$ corresponds to a true source location, $d^{2}$→0 due to the orthogonality condition, and thus 1/$d^{2}$→∞, producing a sharp peak.

The localization ambiguity surface for FDSL-MUSIC is obtained by incoherently averaging over N difference frequency values, as given by (21).

(21) $$B_{\mathrm{FDSL}\text{-}\mathrm{MUSIC}}\left(x_{t}\right)=\frac{1}{N}\sum_{n=1}^{N}\frac{1}{w^{H}\left(x_{t},∆\omega_{n}\right)E_{n}E_{n}^{H}w\left(x_{t},∆\omega_{n}\right)}$$

## 4. Simulation Results and Analysis

**Figure 4:**
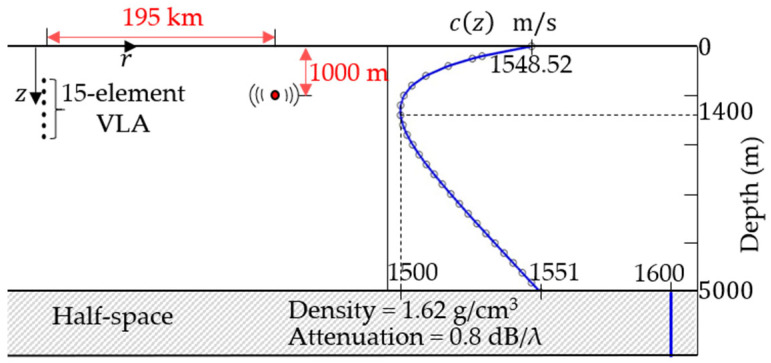
Deep-sea simulation environment with Munk sound speed profile.

The deep-sea Munk environment was adopted for the simulation, with the sound speed profile, environmental parameters, and source location information illustrated in Figure 4. Assuming the number of sources is known a priori, a single-source scenario (D=1) is adopted as a baseline to evaluate the localization performance of the proposed methods. The source-to-array range is 195 km, the source depth is 1000 m, and the depth of the sound speed minimum is 1400 m. The source emitted a wideband signal with a frequency band of 250–350 Hz, which was received by a 15-element vertical line array (VLA) with 70 m uniform spacing, deployed at depths ranging from 580 m to 1560 m. The waveguide water-column depth was set to 5 km. Although the baseline study [12] considered the difference frequency $∆\omega \in \left\{1,\;2,\;3,\;4,\;5\right\}$ Hz, the reduced set $\left\{1,\;3,\;5\right\}$ achieved nearly the same performances as the full sweep in our evaluations. Thus, $∆\omega \in \left\{1,\;3,\;5\right\}$ Hz was adopted to reduce the computational complexity while preserving performance. Given the frequency resolution set to δω=1 Hz, the calculated values of K for ∆ω of 1, 3, and 5 Hz are 100, 98, and 96, respectively. The ocean surface was modeled as a pressure-release boundary, and the sound speed in the acoustic–elastic half-space is 1600 m/s.

To characterize the noise level across all array elements, the array signal-to-noise ratio (SNR) is defined as

(22) $$\mathrm{SNR}=10{log}_{10}\frac{\sum_{m=1}^{M}\sum_{q=1}^{Q}{\left|P_{m}\left(\omega_{q}\right)\right|}^{2}}{Q\sum_{m=1}^{M}\sigma_{m}^{2}}$$

where $P_{m}\left(\omega_{q}\right)$ denotes the magnitude at the q-th frequency bin corresponding to the received signal bandwidth of the m-th element, Q is the number of frequency bins, $\sigma_{m}^{2}$ is the noise power at the m-th element, and $\sigma^{2}=1/M\sum_{m=1}^{M}\sigma_{m}^{2}$ is the average noise power of the array.

**Figure 5:**
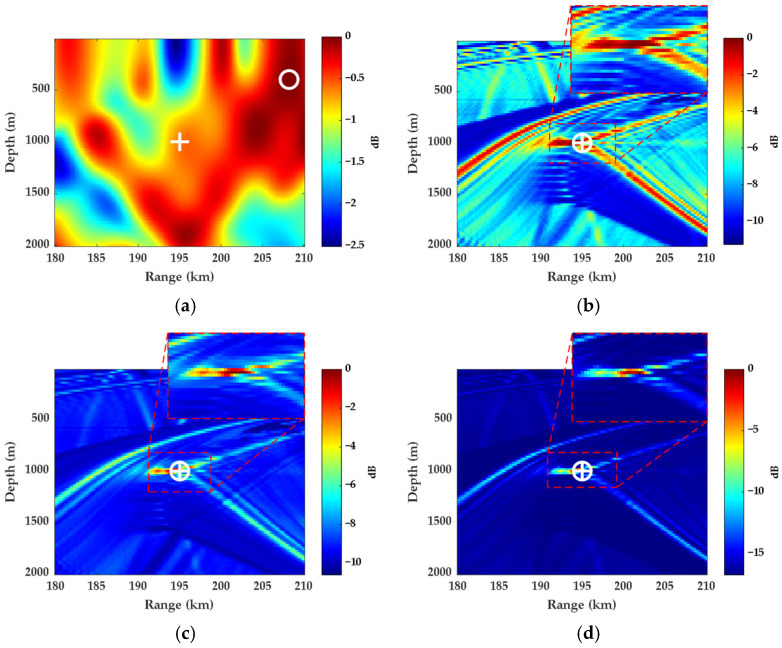
Localization ambiguity surfaces at SNR = 0 dB with difference frequencies of 1, 3, and 5 Hz: (**a**) FDMFP; (**b**) FDSL; (**c**) FDSL-MVDR; (**d**) FDSL-MUSIC.

Figure 5 compares the localization performance of FDMFP, FDSL, FDSL-MVDR, and FDSL-MUSIC by illustrating their ambiguity surfaces computed using Equations (8), (12), (15) and (21), respectively. The symbols “+” and “o” indicate the true and estimated source locations, respectively.

Figure 5 presents the localization ambiguity surfaces of the four methods at SNR = 0 dB. As observed in Figure 5a, FDMFP exhibits numerous spurious peaks on the ambiguity surface due to the absence of caustic phase compensation. The main lobe significantly deviates from the true source location, with the estimated position at a range of 208 km and a depth of 400 m, resulting in localization failure. In contrast, Figure 5b–d demonstrate that FDSL effectively compensates for caustic phase errors through difference-frequency processing of the replica field. All three FDSL methods achieve accurate localization, with the estimated position at a range of 195 km and a depth of 1000 m, matching the true source location. Among these methods, conventional FDSL exhibits relatively high sidelobe levels across the search space. FDSL-MVDR achieves improved sidelobe suppression with a narrower main lobe compared to conventional FDSL. FDSL-MUSIC produces a well-defined main peak with lower background noise, showing better spatial resolution. These results validate the effectiveness of the proposed adaptive FDSL methods, which demonstrate improved performance over conventional FDSL in terms of spatial resolution and sidelobe suppression for deep-sea long-range source localization scenarios.

**Figure 6:**
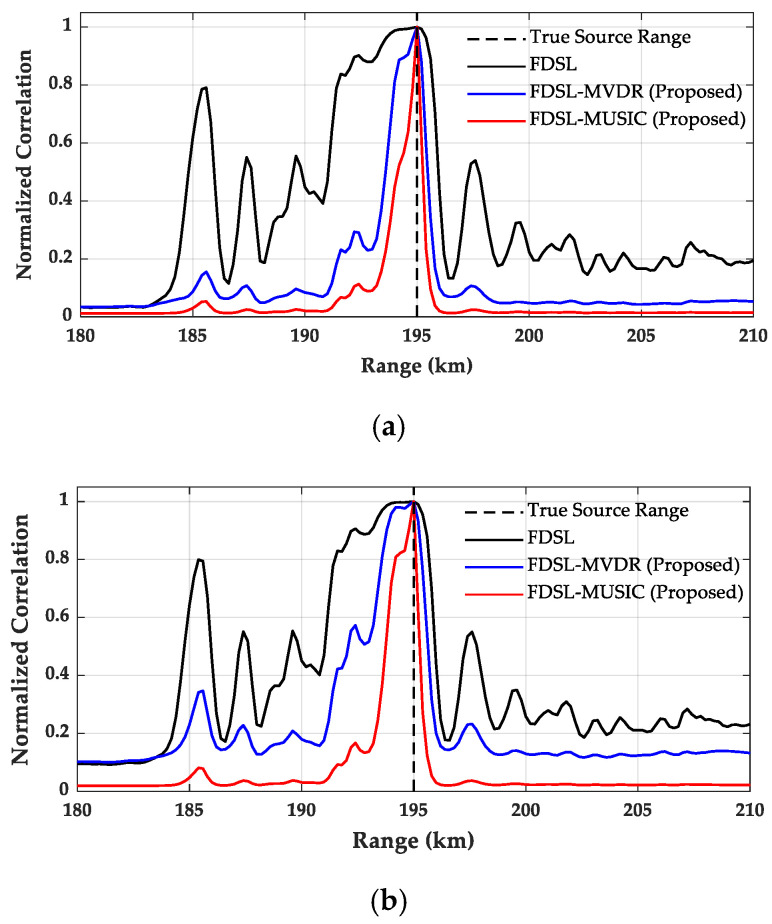
Normalized range profiles at the true source depth (1000 m) for FDSL, FDSL-MVDR, and FDSL-MUSIC. (**a**) SNR = 10 dB; (**b**) SNR = 0 dB.

To evaluate and compare the localization performance and resolution characteristics of FDSL, FDSL-MVDR, and FDSL-MUSIC, normalized range profiles were extracted at the true source depth of 1000 m. Figure 6 presents the comparative analysis of these three methods under SNR conditions of 10 dB and 0 dB, respectively.

As illustrated in Figure 6a, at SNR = 10 dB, all three methods successfully identify the source at 195 km, yet they exhibit substantial differences in spatial resolution and sidelobe suppression capabilities. Conventional FDSL exhibits a broad main lobe accompanied by prominent sidelobes distributed throughout the search range, with multiple spurious peaks emerging across the 180–200 km region. This creates the highest background noise level among the three methods and poses potential ambiguity in source localization. FDSL-MVDR demonstrates significantly reduced background levels and a markedly narrower main lobe compared to conventional FDSL, though some low-amplitude residual artifacts remain visible near the source region. FDSL-MUSIC demonstrates superior performance, featuring the sharpest main peak with the lowest background noise level.

As shown in Figure 6b, at SNR = 0 dB, the localization performance of all three methods degrades compared with the 10 dB case, as indicated by broadened main lobes and increased background noise levels. Nevertheless, FDSL-MUSIC continues to outperform the other two methods, maintaining the sharpest main peak and the lowest background level, providing the most reliable localization result under this low-SNR condition.

**Figure 7:**
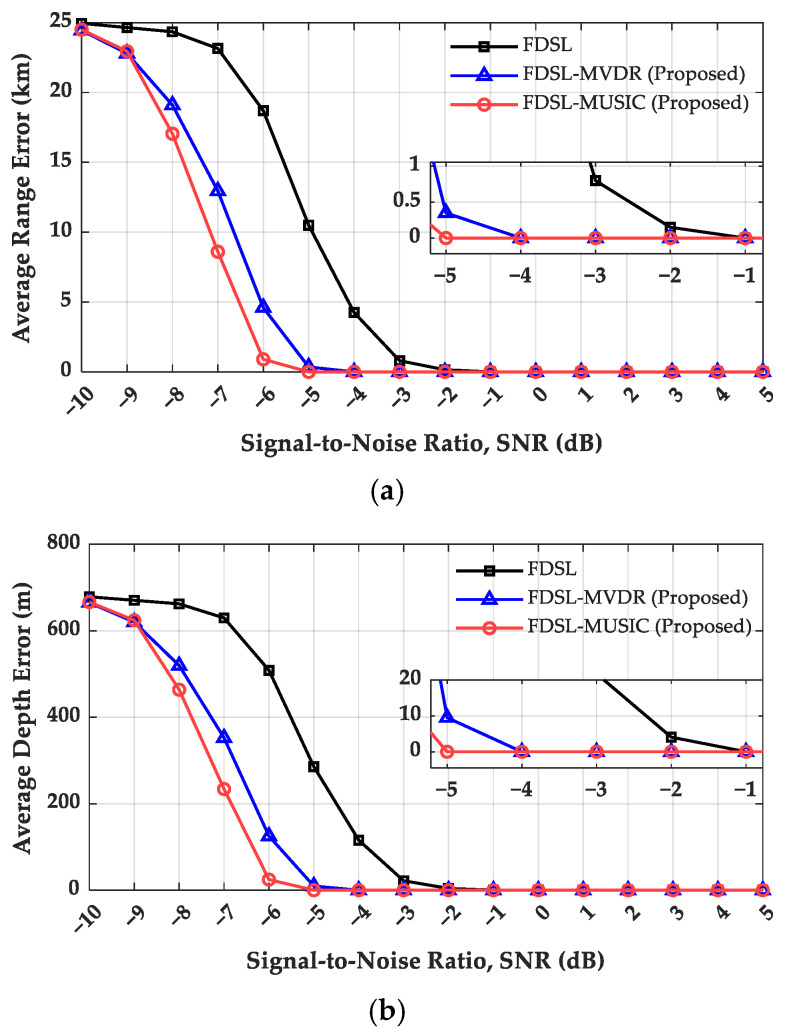

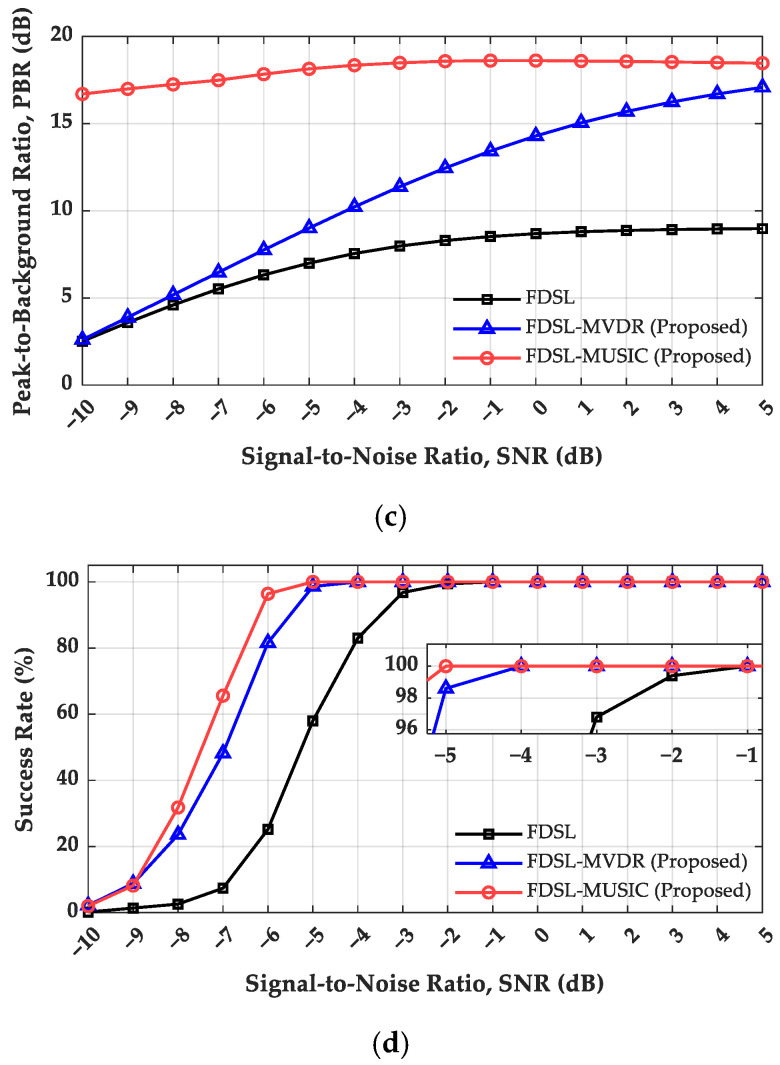
FDSL performance comparison under different SNR conditions. (**a**) Average range error; (**b**) average depth error; (**c**) peak-to-background ratio; (**d**) success rate.

To comprehensively evaluate the performance of FDSL, FDSL-MVDR, and FDSL-MUSIC under varying SNR conditions, Monte Carlo simulations were conducted with 500 independent trials at each SNR level ranging from −10 dB to 5 dB. Figure 7 presents comprehensive performance metrics across these SNR levels, with four key performance indicators evaluated:

(a) Localization Error: The absolute differences between the true and estimated positions in range and depth.
(b) Peak-to-Background Ratio (PBR): Defined as the ratio of the ambiguity surface peak value to the average background level [8], as expressed in (23):
  (23) $$\mathrm{PBR}=10{log}_{10}\left(\frac{E-\beta }{\beta }\right)$$
  where E denotes the peak energy and β represents the average background energy.
(c) Success Rate: Localization is considered successful when both the range and depth errors between the estimated and true positions are within three grid points [10].

To comprehensively evaluate the performance of the three FDSL methods under varying SNR conditions, Monte Carlo simulations were conducted with 500 independent trials at each SNR level ranging from −10 dB to 5 dB in 1 dB intervals.

Figure 7a,b reveal the range and depth error characteristics, identifying distinct SNR thresholds for achieving zero-error localization. Conventional FDSL requires an SNR of −1 dB or higher to attain accurate zero-error localization in both range and depth dimensions. FDSL-MVDR exhibits improved performance, achieving zero errors at SNR = −4 dB, representing a 3 dB improvement over conventional FDSL. FDSL-MUSIC demonstrates optimal performance, attaining accurate localization at SNR = −5 dB, thereby providing a 4 dB improvement relative to conventional FDSL.

Figure 7c illustrates the PBR evolution, revealing distinct performance characteristics among the three methods. FDSL-MUSIC consistently maintains the highest PBR values throughout the entire SNR range, sustaining approximately 17–18 dB over the tested SNR range, thereby reflecting superior peak clarity and background suppression. FDSL-MVDR exhibits a pronounced upward trend, with PBR values substantially increasing from approximately 3 dB at SNR = −10 dB to approximately 17 dB at SNR = 5 dB. In contrast, conventional FDSL demonstrates limited PBR growth, with values increasing from approximately 3 dB to only 9 dB across the SNR range, remaining substantially lower than both adaptive methods.

Figure 7d presents the localization success rate evolution, validating the performance trends observed in the error analysis. FDSL-MUSIC achieves an approximately 100% success rate at SNR = −5 dB, FDSL-MVDR attains complete reliability at SNR = −4 dB, while conventional FDSL requires an SNR of −1 dB or higher for 100% success. These results demonstrate that even with a 15-element array configuration, the proposed adaptive methods, particularly FDSL-MUSIC, enable reliable deep-sea long-range source localization under low-SNR conditions.

In practical applications, environmental parameters such as sound speed profile, water depth, seabed properties, and array geometry are difficult to obtain accurately, resulting in inevitable environmental mismatch between the replica field used by localization methods and the measured field in actual acoustic propagation. Environmental mismatch tolerance represents a significant advantage of FDSL over conventional matched-field processing. Although the proposed adaptive FDSL methods demonstrate superior performance under perfectly matched conditions, their robustness under realistic environmental uncertainties requires systematic evaluation. Whether the inherent mismatch tolerance of conventional FDSL is preserved within the adaptive framework, and the effectiveness of adaptive enhancement under imperfect environmental knowledge, must be validated through representative mismatch scenarios.

To assess the robustness of the proposed methods, four typical mismatch scenarios were evaluated: sound speed profile (SSP) bias introduced as constant offsets of ±2 and ±5 m/s, water depth mismatch of ±100 and ±200 m, seabed attenuation mismatch ranging from 0.1 to 1.5 dB/λ, and array element position mismatch modeled as random depth perturbations Δz ~ *𝒩* (0, $\sigma^{2}$) with σ = 1, 5, and 10 m. Table 1, Table 2, Table 3 and Table 4 report the range error, depth error, and PBR under these mismatch conditions, based on 500 Monte Carlo trials conducted at SNR = 0 dB with the source positioned at a range of 195 km and a depth of 1000 m to ensure statistical reliability.

Based on the simulation results, the FDSL-MUSIC method generally achieves the highest PBR. As shown in Table 1, Table 2 and Table 3, regarding environmental adaptability, all three methods exhibit strong robustness against mismatches in SSP, water depth, and seabed attenuation. The depth estimation error consistently remains zero, while the maximum range error is 0.4 km, corresponding to only two range grid intervals. As shown in Table 4, regarding array element position mismatch, the depth error is 20 m even under the large deviation condition (σ=10 m), which is equivalent to a single depth grid interval. It is well established that the high-resolution subspace-based MUSIC method relies on precise array manifold modeling. Consequently, environmental mismatches or geometric deviations may lead to degradation in localization performance. However, the proposed FDSL-MVDR and FDSL-MUSIC methods demonstrate numerical stability across the examined ranges of SSP, water depth, seabed attenuation, and array element position mismatches, effectively preserving the inherent environmental robustness of the conventional FDSL method.

In terms of computational efficiency, the two proposed adaptive FDSL methods are comparable to the conventional FDSL method. By utilizing pre-computed replica fields, the dominant computational burden for all three methods resides in the grid search process. The adaptive matrix operations, specifically matrix inversion for MVDR and eigendecomposition for MUSIC, are executed only once per difference frequency. Given the few-element array configuration, the additional overhead imposed by these low-dimensional matrix operations is negligible compared to the extensive grid search. Consequently, the computational costs of the three methods are similar for real-time applications.

## 5. Conclusions

This paper proposes the FDSL-MVDR and FDSL-MUSIC methods to address resolution limitations in deep-sea long-range source localization with a few-element array.

Numerical simulations in a canonical deep-sea Munk environment (195 km range, 1000 m depth) using a 15-element vertical line array validate the effectiveness of the proposed methods. The results demonstrate that the proposed adaptive methods achieve enhanced spatial resolution and superior sidelobe suppression. Specifically, the adaptive methods, particularly FDSL-MUSIC, significantly outperform conventional FDSL under low-SNR conditions. Furthermore, the robustness of the proposed methods is verified against typical environmental uncertainties.

Future research directions include experimental validation with sea trial data and extension to multi-source scenarios.

## Figures and Tables

**Table 1:** Performance comparison under different SSP mismatch scenarios.

Method	Metric	0 m/s (Matched)	+2 m/s	−2 m/s	+5 m/s	−5 m/s
FDSL	Range Error (km)	0	0	0.200	0.201	0.400
	Depth Error (m)	0	0	0	0	0
	PBR (dB)	8.68	8.67	8.67	8.67	8.69
FDSL- MVDR	Range Error (km)	0	0	0.200	0.204	0.400
	Depth Error (m)	0	0	0	0	0
	PBR (dB)	14.28	14.64	14.70	14.63	14.74
FDSL- MUSIC	Range Error (km)	0	0	0.200	0.2	0.400
	Depth Error (m)	0	0	0	0	0
	PBR (dB)	18.61	17.40	17.82	17.32	17.20

**Table 2:** Performance comparison under different water depth mismatch scenarios.

Method	Metric	0 m (Matched)	−100 m	+100 m	−200 m	+200 m
FDSL	Range Error (km)	0	0	0	0.125	0
	Depth Error (m)	0	0	0	0	0
	PBR (dB)	8.68	8.65	8.69	8.23	8.69
FDSL- MVDR	Range Error (km)	0	0	0	0.2	0
	Depth Error (m)	0	0	0	0	0
	PBR (dB)	14.28	14.76	14.58	13.32	14.59
FDSL- MUSIC	Range Error (km)	0	0	0	0.082	0
	Depth Error (m)	0	0	0	0	0
	PBR (dB)	18.61	18.16	17.12	17.49	18.09

**Table 3:** Performance comparison under different seabed attenuation mismatch scenarios.

Method	Metric	0.1 dB/λ	0.2 dB/λ	0.4 dB/λ	0.8 dB/λ (Matched)	1.2 dB/λ	1.5 dB/λ
FDSL	Range Error (km)	0.137	0.005	0	0	0	0
	Depth Error (m)	0	0	0	0	0	0
	PBR (dB)	8.71	8.71	8.70	8.68	8.68	8.68
FDSL- MVDR	Range Error (km)	0.035	0.001	0	0	0	0
	Depth Error (m)	0	0	0	0	0	0
	PBR (dB)	13.63	13.64	14.65	14.28	14.68	14.69
FDSL- MUSIC	Range Error (km)	0.054	0	0	0	0	0
	Depth Error (m)	0	0	0	0	0	0
	PBR (dB)	17.32	17.36	17.48	18.61	17.67	17.71

**Table 4:** Performance comparison under different array element position mismatch scenarios.

Method	Metric	σ=0 (Matched)	σ=1 m	σ=5 m	σ=10 m
FDSL	Range Error (km)	0	0	0.200	0.200
	Depth Error (m)	0	0	0	20
	PBR (dB)	8.68	8.21	8.11	8.08
FDSL- MVDR	Range Error (km)	0	0	0.200	0.200
	Depth Error (m)	0	0	0	20
	PBR (dB)	14.28	13.49	13.57	13.51
FDSL- MUSIC	Range Error (km)	0	0	0.200	0.200
	Depth Error (m)	0	0	0	20
	PBR (dB)	18.61	17.89	17.78	17.45

## Data Availability

The original contributions presented in this study are included in the article. Further inquiries can be directed to the corresponding author.
